# Comparison of three interventional approaches to prevent ventilator-associated pneumonia in intensive care units (ICUs): A clinical trial study

**DOI:** 10.5339/qmj.2021.21

**Published:** 2021-08-23

**Authors:** Nader Zarinfar, Ehsanollah Ghaznavi-Rad, Behnam Mahmoodiyeh, Azita Reyhani

**Affiliations:** ^1^Department of Infectious Disease, School of Medicine, Infectious Disease Research Center, Amiralmomenin Hospital, Valiasr Hospital, Arak University of Medical Sciences, Arak, Iran; ^2^Molecular Research Center, Faculty of Medicine, Arak University of Medical Sciences, Arak, Iran; ^3^Department of Anesthesiology and Critical Care, Arak University of Medical Science Arak, Iran; ^4^Department of Infectious Disease, School of Medicine, Arak University of Medical Science, Arak, Iran E-mail: Dr.areyhani@gmail.com

**Keywords:** ventilator-associated pneumonia, prevention, VAP prevention bundle measures

## Abstract

Background: Ventilator-associated pneumonia (VAP) is an infectious pulmonary disease that develops after 48 hours of ventilation. To date, several methods have been proposed to reduce VAP occurrence, such as the VAP prevention bundle, which involves raising the head of the bed, reducing sedation, avoiding deep vein thrombosis, and preventing peptic ulcer in the gastrointestinal system. The purpose of this study was to evaluate the role of personnel in hand washing, case airway suctioning, and systematic monitoring in the prevention of VAP.

Methods: In the current clinical trial, 129 patients hospitalized and intubated at Vali-e-Asr Hospital ICU in Arak, Iran, were included in the study and randomized to one of the three VAP prevention methods: group A, VAP prevention bundle measures; group B, group A measures plus washing of patients’ mouth with 0.12% chlorhexidine and suction of secretion every six hours; and finally group C, group B measures plus 72-hour suction package. Demographic information, VAP diagnosis, and outcome of each patient were recorded in the special checklist.

Results: The age of the patients ranged from 18 years to 93 years with a mean of 54.6 ± 21.8 years. There was no significant difference in age, sex, Clinical Pulmonary Infection Score (CPIS), and Glasgow Coma Scale (GCS) between the three groups. However, there is a significant relationship between chest X-ray (CXR) index and pneumonia in the three groups (*p* < 0.05). The prevalence of pneumonia is generally seen to be higher in patients who were local, diffuse, or patchy than those who had no infiltration (*p* < 0.05).

Conclusion: This study showed that the application of VAP prevention bundle measures, mouthwash with chlorhexidine, personnel hand washing, airway suctioning, and systematic monitoring is an efficient approach to the prevention of VAP in ICUs.

## Introduction

Ventilator-associated pneumonia (VAP) is one of the very complicated conditions in the ICUs that generally occurs 24–72 hours after endotracheal intubation, with symptoms such as high body temperature, altered count of white blood cells, presentation of chest infiltration based on radiography results, etc.^1-3^ VAP contributes to nearly 50% of all acquired pneumonia cases in hospitals.^3,4^ This condition is one of the most common hospital-related nosocomial infections, especially in subjects that received mechanical ventilation in the ICU.^5^ In addition, epidemiological studies have shown that the prevalence of VAP was 5.0–9.6%, with a high mortality rate of 23.6–47.5% worldwide.^6-8^ Given the fact that precise sterilization of respiratory equipment and also advances in pharmaceutical sciences have reduced the incidence of VAP, the aforementioned condition is associated with a high mortality rate, particularly when this condition is triggered by high-risk pathogens and is a confounding problem for infectious disease specialists.^7^ One of the most effective issues that play a crucial role in the management of infection in VAP cases is the rapid detection of infected subjects and selection of suitable therapeutic approaches.^7,8^

In the previous scientific investigations, some approaches have been proposed to reduce the occurrence of VAP, such as VAP prevention bundle, including raising the patient’s bed by 30–45 degrees, reducing patient sedation, and extubating with no complication, avoiding deep vein thrombosis, and even preventing peptic ulcer in the gastrointestinal tract.^9,10^ In addition, more preventive measures such as hand washing by clinicians, frequent mouthwashing, patient baths, sublingual discharge suctioning, use of special types of endotracheal tubes, increased endotracheal tube pressure, and probiotic use play an important role in the prevention of VAP.^11-13^ The development of a cost-effective model to reduce VAP in the nosocomial infection control program appears to be important. Furthermore, this current study aims to evaluate the effectiveness of some affordable measures without careful supervision, such as washing the patient’s mouth and staff’s hands and suctioning the patient’s subglottal discharge in the prevention of VAP. This approach can be integrated as a post-research model into a national nosocomial infection control program, thereby systematically reducing VAP and decreasing costs and related disability and mortality.

## Materials And Methods

### Patients and study design

Current clinical trial study was approved by the Department of Infectious Diseases at Vali-e-Asr Hospital, Arak University of Medical Sciences, Arak, Iran. Additionally, the agreement of the Ethics Committee (Number: IR.ARAKMU.REC.1396.270) of the university was obtained, which was conducted from September 2017 to February 2018. In the present double-blind clinical trial study, 129 patients admitted to the ICU of Vali-e-Asr Hospital (surgery, neurology, and neurosurgery) in Arak were enrolled after obtaining written informed consent. Intubated patients in all three ICUs of neurology, neurosurgery, and surgery departments were randomly divided into three groups of VAP prevention: **group A**, only under the VAP prevention bundle measures including staff hand washing (prior to any action), case suctioning, and systematical monitoring, raising the patient’s head by 30–45 degrees, decreasing the patient’s sedation rate, evaluating the patient for extubation, avoiding deep vein thrombosis (in the form of intravenous anti-coagulation use and in cases of mechanical restriction with compressive socks), and preventing peptic ulcer (using proton pump inhibitor drugs or H2 receptor blockers); **group B**, group a measures plus patient mouthwash with chlorhexidine twice daily and repeated airway suctioning of ventilated patient at least every 6 hours; and **group C**, group B measures plus 72-hour suction package.

Exclusion criteria included the use of immunosuppressive drugs, pregnancy, occurrence of catheter-related infection, endocarditis, urinary tract infection, and other infection conditions.

Clinical Pulmonary Infection Score (CPIS) is one of the criteria for the diagnosis of pneumonia with six indices, each of which scores from 0 to 2 and where a score of more than 6 is considered pneumonia. In this study, CPIS more than 6 was considered as the criterion of VAP diagnosis. In addition, sputum culture (agar culture and differential tests) was performed for the definitive diagnosis of VAP. Demographic information of cases was documented in special forms. Each patient was given a checklist completed on a regular basis that includes evaluation of bed angle, suctioning rate, evaluation of patient sedation or consciousness, intravenous thrombosis, temperature, leukocytosis, and leukopenia (by blood test).

### Statistical analysis

For the statistical analysis, we used the SPSS software, Version 22.0 (SPSS Inc., Chicago, IL, USA). The aforementioned purpose was performed through descriptive statistics, unpaired Student’s t-test, x^2^ test, and one-way ANOVA test, and *p* < 0.05 was accepted as a statistically significant difference. Student’s t-test was used to determine the VAP rate and comparison of CPIS, Glasgow Coma Score, and CXR index based on the three groups between male and female, whereas one-way ANOVA was used in the abovementioned A, B, and C groups. Correlation analysis was also used to evaluate any significant association between age, gender, etc. and the abovementioned items. In addition, the power of the analysis was evaluated by using G*Power software to reach at least β = 85%.

## Results

### Patients’ demographic descriptive analysis

In present study, three approaches to the prevention of VAP occurrence were evaluated. Moreover, 43 (33.3%) cases were studied in each group (129 patients). Patient age ranged from 18 years to 93 years with a mean age of 54.6 ± 21.8 years. The mean age of patients in group A was 60.4 ± 21.2 years, in group B was 53.5 ± 22.9 years, and in group C was 50 ± 20.3 years. Based on the statistical analysis, the observed differences were random and were not significant (*p* = 0.08, f = 2.583). In group A, 48.8% were male and 51.2% were female, while in group B, 60.5% were male and 39.5% were female, and in group C, 44.2% were male and 55.8% were male. Statistically, the differences mentioned above were not significant (*p* = 0.298).

### Evaluation on the role of age and gender in the presence of VAP

The mean age of patients with pneumonia was 49.63 ± 23.23, while the mean age of patients without pneumonia was 57.41 ± 20.55 years. Although it was found that the mean age of patients with pneumonia was lower than the mean age of patients without pneumonia, this disparity was marginally significant at 0.05 level of error (*p* = 0.052).

Table 5 presents the gender frequency of patients in terms of presence or absence of pneumonia (based on the chi-square test). Statistically, the observed difference was not statistically significant at 0.05 level of error (*p* = 0.365).

### Evaluation on the rate of VAP and comparison of clinical pulmonary infection score, Glasgow Coma score, and chest x-ray index based on the three groups

Table 1displays the mean CPIS of patients in three groups and compared using a one-way analysis of variance. The mean CPIS was 4.88 ± 2.59 of patients in group A, 6.14 ± 2.47 in group B, and 5 ± 3.02 in group C. Statistically, these differences were marginally significant (*p* = 0.062, f = 2.837). Table 2 describes the mean of Glasgow Coma Scale (GCS) in three groups and compared using a one-way variance analysis. Based on statistical analysis, these differences were not significant (*p* = 0.073, f = 2.678). Table 3 presents the chest X-ray (CXR) index of patients in three groups and compared with chi-square test, and these differences were not significant (*p* = 0.277). Table 4 indicates the frequency of pneumonia in the patients examined in three groups and compared with the chi-square test (the difference was not significant, *p* = 0.789). The mean GCS for all subjects was 7.64 ± 3.55. Further, the mean GCS for patients with pneumonia was 7.26 ± 3.16, and the mean GCS for patients without pneumonia was 7.86 ± 3.76. Although it is found that the mean GCS of pneumonia patients is lower than the mean GCS of patients without pneumonia, this difference was not statistically significant at 0.05 level of error (*p* = 0.365). Table 6 presents the CXR Index in the studied patients based on pneumonia in three groups and compared with the chi-square test. There was a significant relationship between the CXR Index and pneumonia in three groups at 0.05 levels. It is generally seen that the prevalence of pneumonia was higher in patients who had local, diffuse, or patchy lesions in their radiological studies than in those who had no infiltration (*p* < 0.05).

## Discussion

Cases in the ICUs are at high risk for death not only due to their serious conditions but also due to secondary complication such as VAP.^14^ So far, several studies attempted to examine the effects of various approaches to the prevention of VAP. Anatomical alterations will result in decreased pulmonary volumes and pulmonary capacity following atelectasis and secretion of alveoli.^15,16^ Decreased airway resistance and pressure and pulmonary shunts are all complications of incorrect pulmonary suctioning and other remedies.^17^ In the present study, the percentage of men did not vary significantly from women. It seems that men were more likely to develop pneumonia than women because of higher lung disease and smoking,^18^ but not certain.

The present clinical trial was conducted to compare three intervention methods A, B, and C for the prevention of VAP in ICUs. In the three groups, mean age, sex, GCS, and CPIS were similar (*p*>0.05). Due to the random division of subjects in one of the three prevention methods, expectation of age and sex similarity is acceptable. Although the occurrence of pneumonia decreased from group A to group C, there was no statistically significant difference between the three groups. One of the considerable issues was more measures for group C, but not a significant effect on the reduction of pneumonia. It might be due to the history of antibiotic therapy in some patients connected with the aforementioned result. In other words, it can be argued that VAP prevention bundle measures are an efficient way to prevent VAP. In their cross-sectional study, Sabery and coworkers (2013) investigated the frequency and risk factors for early-onset VAP in ICUs at Kashan University Hospitals, Kashan, Iran.^19^ According to their findings, there was a significant statistical difference between the clinician who inserts a catheter and the positioning of the patient’s head at ≥ 30. Raising the patient’s head by 30–45 degrees is one of the crucial parts of VAP prevention bundle measures, and based on our results, it can be seen that present study results are consistent with the Sabery et al., study. Also, our results are consistent with the study performed by Caserta et al., (2012). In their quasi-experimental study, they showed that bundle efficacy was greater than 90% between health care, VAP prevention bundle measures, mouthwash with chlorhexidine, and subglottic sputum.^20^

In our clinical trial study, some limitations were also confronted. One of the limitations was a few numbers of subjects that participated in the investigation. As we know, more patients need to make a realistic conclusion about the effectiveness of a prevention approach. Also, in present study, there were no precise history of taking antibiotics and consequent related resistance in patients, and this issue may confront our conclusion with some unwanted bias. Also, for examining the effectiveness of prevention approaches, CPIS was used. To date, some studies were conducted to evaluate the efficacy of the CPIS index as a reliable method for diagnosing and evaluating cases with VAP. It has been reported that a CPIS of more than 6 may be linked to the presence of VAP. Papazian et al., (1995) presented that the specificity and sensitivity of CPIS was 85% and 72%, respectively.^21^ Also, they declared that CPIS had a general reliability of 79% for the diagnosis of VAP in patients. As we all know, the aforementioned specificity, sensitivity, and reliability of CPIS were associated with a limited role in the diagnosis and assessment of VAP, but, due to its repeatable and noninvasive nature, CPIS is widely used in clinical studies performed on VAP subjects. Finally, it should be noted that this study had some limitations, including the association between smoking, history of antibiotic therapy, comorbidity, lab data, and the main cause of intubation and the presence of VAP were not addressed. In this regard, future studies need to be established to evaluate the association of the abovementioned factors based on prevention strategies used in the current manuscript.

## Conclusion

Based on the results of the present study, it cannot be concluded that the application of VAP prevention bundle measures, personnel hand washing, discharge suctioning, and systematic monitoring is an effective approach to the prevention of VAP in ICUs; however, this issue needs to be evaluated in large-scale population. This study suggested that mouthwash with chlorhexidine twice daily and repeated airway suctioning of ventilated patient might decrease the risk of VAP in incubated patients.

### Acknowledgment

The current study was supported financially by the Deputy Research of Arak University of Medical Sciences, Arak, Iran. We would like to thank the Vice Chancellor for research for the financial support.

### Authors’ Contributions

Nader Zarinfar and Ehsanollah Ghaznavi-Rad conceived the study; Behnam Mahmoodiyeh and Azita Reyhani analyzed the data; and Nader Zarinfar, Ehsanollah Ghaznavi-Rad, Behnam Mahmoodiyeh, and Azita Reyhani drafted the manuscript. All authors read and approved the final manuscript.

### Competing Financial Interests

The authors declare that there is no conflict of interest.

### Funding

This research received no specific grant from any funding agency in the public.

## Figures and Tables

**Table 1:** CPIS (mean ± SD) of the patients (p = 0.062, f = 2.837)

**CPIS**	**Group**			**All**

	A	B	C	

Mean	4.88	6.14	5.00	5.34

Standard deviation	2.59	2.47	3.02	2.74


**Table 2:** GCS (mean ± SD) of cases in three groups (p = 0.073, f = 2.678)

**GCS**	**Groups**			All

	A	B	C	

Mean	8.63	6.95	7.35	7.64

Standard deviation	4.37	2.75	3.19	3.55


**Table 3:** The CXR index of patients in three groups (p = 0.277)

**CXR Index**	**Groups**		

	A	B	C

Local	14 (32.6%)	11 (25.6%)	8 (18.6%)

Diffuse or patchy	11 (25.6%)	7 (16.3%)	14 (32.6%)

No infiltration	18 (41.9%)	25 (58.1%)	21 (48.8%)

All	42 (100%)	42 (100%)	42 (100%)


**Table 4:** Frequency of pneumonia in the studied patients in three groups (p = 0.789)

**Groups**	**Pneumonia**		**All**

	Positive	Negative	

A	17 (39.5%)	26 (60.5%)	43 (100%)

B	15 (34.9%)	28 (65.1%)	43 (100%)

C	14 (32.6%)	29 (67.4%)	43 (100%)

Total	46 (35.7%)	83 (64.3%)	43 (100%)


**Table 5:** The frequency of gender of the patients in terms of presence or absence of pneumonia (p = 0.365)

**Groups**	**Sex**	**Pneumonia**		**Total**

		Positive	Negative	

A	Male	7 (33.3%)	14 (66.7%)	21 (100%)

	Female	10 (45.5%)	12 (54.5%)	2 (100%)

B	Male	13 (50%)	13 (50%)	26 (100%)

	Female	2 (11.8%)	15 (88.2%)	17 (100%)

C	Male	6 (31.6%)	13 (68.4%)	19 (100%)

	Female	8 (33.3%)	16 (66.7%)	24 (100%)

Total	Male	25 (39.4%)	40 (60.6%)	66 (100%)

	Female	20 (31.7%)	43 (68.3%)	63 (100%)


**Table 6:** The CXR Index in the studied patients based on pneumonia

**Group**	**CXR Index**	**Pneumonia**		**Total**	*p* ** value**

		Positive	Negative		

A	No infiltration	1 (9.1%)	10 (90.9%)	11 (100%)	0.005

	Diffuse or patchy	6 (33.3%)	12 (66.7%)	18 (100%)	

	Local	10 (71.4%)	4 (28.6%)	14 (100%)	

B	No infiltration	0	5 (100%)	5 (100%)	0.030

	Diffuse or patchy	8 (29.6%)	19 (70.4%)	27 (100%)	

	Local	7 (63.6%)	4 (36.4%)	11 (100%)	

C	No infiltration	1 (6.7%)	14 (93.3%)	15 (100%)	0.029

	Diffuse or patchy	10 (47.6%)	11 (52.4%)	21 (100%)	

	Local	3 (42.9%)	4 (57.1%)	7 (100%)	

